# Supplementary material for: Abbreviated Versus Multiparametric Prostate MRI in Active Surveillance for Prostate-Cancer Patients: Comparison of Accuracy and Clinical Utility as a Decisional Tool
Source: Diagnostics (Basel). 2023 Feb 4;13(4):578. doi: 10.3390/diagnostics13040578 (PMC9955028; doi:10.3390/diagnostics13040578)
Supplement: Supplementary file 1 [file diagnostics-13-00578-s001.zip › Supplementary Table S1 - MRI Acquisition Parameters.pdf]

Supplementary Table S1 – Acquisition parameters of multiparametric prostate magnetic resonance imaging.

	**DWI single sequence**	**TSE-T2WI**	**DCE**
**Sequence**	SS-EPI	TSE	THRIVE
**Weighting**	DWI	T2	T1
**Acquisition plane**	Transverse	Transverse/coronal/sagittal	Transverse
**TR (ms)**	4458	4727/3076/3714	3.4
**TE (ms)**	74	80/80/80	1.77
**Echo train length**	-	8/16/16	-
**EPI factor**	75	-	-
**Half scan factor**	0.62	no	no
**FOV (mm x mm)**	200 x 200	180x180/180x180/180x180	200x200
**Acquisition voxel size**	2 x 2 x 3	0.6 x 0.6 x 3	1.2 x 1.2 x 4
**Reconstruction voxel size (mm x mm x mm)**	1.4 x 1.4 x 3	0.45 x 0.45 x 3	0.62 x 0.62 x 4
**Slice thickness (mm)**	3	3/3/3	4
**Number of slices**	20	24/20/20	20
**Interslice gap (mm)**	0	0/0/0	0
**b-values (s/mm^2)/number of excitations**	0/1, 1000/2*, 1500/3, 2000/5**	-	-
**Number of excitations**	-	2/1/1	1
**Fat saturation**	SPAIR	-	Spectral fat saturation
**Parallel imaging (x acceleration factor)**	SENSE x 2	SENSE x 2.5/1/1	SENSE x2
**Acquisition time (min)**	7.03	5.4/4.3/5.1	Total acquisition time 6 (0.14 x 44 acquisitions)

DWI = diffusion-weighted imaging; TSE-T2WI = turbo spin echo T2-weighted imaging; DCE = dynamic contrast-enhanced; THRIVE = T1-weighted high-resolution isotropic volume examination; SS-EPI = single-shot echoplanar imaging; TR = time of repetition; TE = time of echo; EPI = echo planar imaging; FOV = field of view; SENSE = sensitivity encoding; SPAIR = spectral adiabatic inversion recovery; * used to calculate the apparent diffusion coefficient map; ** used to provide b = 2000 s/mm^2 images
