# Supplementary material for: Abbreviated Versus Multiparametric Prostate MRI in Active Surveillance for Prostate-Cancer Patients: Comparison of Accuracy and Clinical Utility as a Decisional Tool
Source: Diagnostics (Basel). 2023 Feb 4;13(4):578. doi: 10.3390/diagnostics13040578 (PMC9955028; doi:10.3390/diagnostics13040578)
Supplement: Supplementary file 1 [file diagnostics-13-00578-s001.zip › Supplementary Table S2 - NCCN Risk Stratification version 2-2019.pdf]

Supplementary Table S2 – NCCN Risk Stratification (version 2.2019)

<table>
<tr><th>Risk group</th><th colspan="3">Clinical/pathological features</th></tr>
<tr><td>Very low</td><td colspan="3">• T1c AND
• Grade Group 1 AND
• PSA < 10 ng/mL AND
• Fewer than 3 prostate biopsy fragments/cores positive with ≤ 50% cancer in each fragment / core AND
• PSA density < 0.15 ng/mL/g</td></tr>
<tr><td>Low</td><td colspan="3">• T1-T2a AND
• Grade Group 1 AND
• PSA < 10 ng/mL</td></tr>
<tr><td rowspan="2">Intermediate</td><td rowspan="2">Has no high- or very-high-risk features and has one or more intermediate risk factors (IRF):
- T2b-T2c
- Grade group 2 or 3
- PSA 10–20 ng/mL</td><td>Favorable intermediate</td><td>• 1 IRF and
• Grade Group 1 or 2 and
• <50% biopsy cores positive</td></tr>
<tr><td>Unfavorable intermediate</td><td>• 2 or 3 IRF and/or
• Grade Group 3 and/or
• ≥50% biopsy cores positive</td></tr>
<tr><td>High</td><td colspan="3">• T3a OR
• Grade Group 4 or Grade Group 5 OR
• PSA >20 ng/mL</td></tr>
<tr><td>Very high</td><td colspan="3">• T3b-T4 OR
• Primary Gleason pattern 5 OR
• >4 cores with Grade Group 4 or 5</td></tr>
</table>
