# Supplementary figures and images for: Abbreviated Versus Multiparametric Prostate MRI in Active Surveillance for Prostate-Cancer Patients: Comparison of Accuracy and Clinical Utility as a Decisional Tool
Source: Diagnostics (Basel). 2023 Feb 4;13(4):578. doi: 10.3390/diagnostics13040578 (PMC9955028; doi:10.3390/diagnostics13040578)

Figure S1.

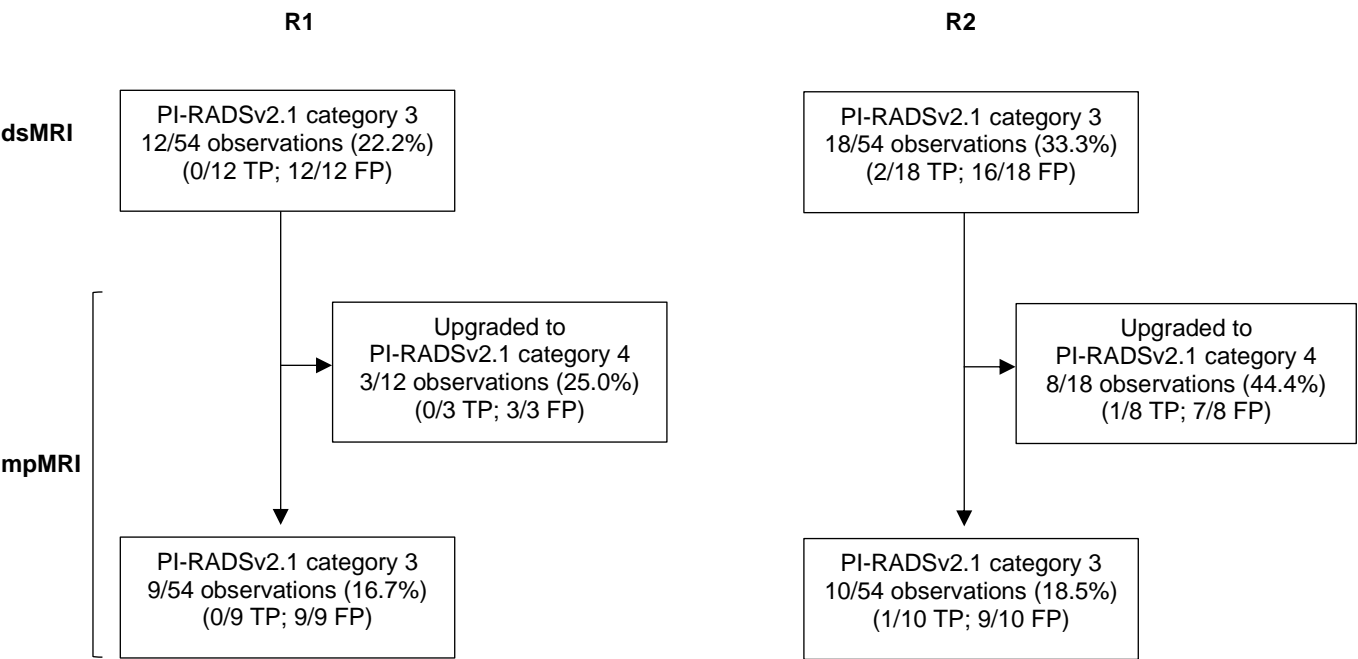

Supplement: Supplementary file 1 [file diagnostics-13-00578-s001.zip › Supplementary Figure S1 - PI-RADS 3.pdf]
